# New paradigm in implant osseointegration

**DOI:** 10.1186/1746-160X-2-19

**Published:** 2006-06-30

**Authors:** Ulrich Joos, Ulrich Meyer

**Affiliations:** 1 Klinik und Poliklinik für Mund-, Kiefer- und Gesichtschirurgie, Universitätsklinikum, Westfälische Wilhelms-Universität, Münster, Waldeyerstr. 30, 48129 Münster, Germany; 2 Klinik für Kiefer- und Plastische Gesichtschirurgie, Westdeutsche Kieferklinik, Moorenstrasse 5, 40225 Düsseldorf, Germany

## Abstract

During the last years, implant dentistry has seen an dramatic increase as a treatment option in oral rehabilitation. This is based to a large extent on scientific advances and clinical improvements in implantology. The extension of indications has broadened the opprtunities to rehabilitate patients that were formerly considered to posess restricted indications to place implants. Additionally, patient desires (high aesthetic demands, fast prosthetic rehabilitation) were placed more in focus, resulting in new approaches in implant dentistry. As a result, the scientific and clinical community has reached high standards and at the same time has founded the basis for new opportunities in implantology. The advances are mirrored by a high number of high quality scientific papers, published in conventional and open-access journals. A major shift has thereby been observed in the understanding of implant healing, leading the basis for new implant systems that allow fast rehabilitation protocols. The term ossseointegration needs a new understanding since an immediate osseointegration state can be present under distinct conditions.

Osseointegration is commonly defined as a direct and stable anchorage of an implant by the formation of bony tissue without growth of fibrous tissue at the bone-implant interface. The term that was initially defined by BRANEMARK et al.[1] as a direct bone-to-implant contact was later on defined on a more functional basis as a direct bone-to-implant contact under load. A defining morphological feature of osseointegration is that osteoblasts and mineralized matrix contacts the implant surface even when loads are applied. In contrast, failure of osseointegration or a disintegration of a formerly stable anchored implant can be conceptualized as a failure of the mineralized extracellular matrix directly attached to the artificial surface, since a mechanically competent implant/bone bond is dependent on an intact mineralized interface structure. Although the bone's capability of dental implant osseointegration has routinely been utilised, the specific mechanisms for the emergence and maintenance of peri-implant bone under functional load was for a long time not identified in detail. Recent research has now shown that osseointegration can not be considered in the traditional sense. Instead of understanding osseointegration as a predetermined time scale event or an end-state of implant healing, osseointegration can now be conceptualised as a highly dynamic process of the all time emergence and maintenance of peri-implant bone. The clinical fate of implants is not dependant on the implant healing time but instead it is dependant on multiple parameters, all influencing the dynamic events that happen in bone. It is now accepted that both aspects of osseointegration, maintenance of present bone (remodelling) and new bone formation (modelling), determine the fate of implant healing[2].

The advances in basic scientific and clinical knowledge are reflected by the high number of high quality papers that are published in traditional and open access journals. The 'intra-interdisciplinary' thinking of medical practitioners and scientists is improved when having access to the scientific literature[3]. The extension of knowledge and the fast alteration of clinical decision making, based to a great extent on the transformation of basic scientific results into practical treatment options, can be examplified by recent alterations in pratical implant dentistry.

The maintenance and emergence was known to be dependent on the extent of surgical trauma directly at insertion as well as through load-related deformations under implant load, especially when immediate or early loading protocols are applied. The initial mode of osseointegration critically depends, therefore, to a greater extent on the geometry of the implant system as well as on the state of cells and matrix at the surface of the artificially created implant site. Considering the recent publication data, it has not yet been convincingly shown on an ultrastructural level that the surgical procedure, if properly performed, is accompanied by a disturbance of cell activity at the surface of the implant bed, nor with a disintegration of bone minerals. The extent of bone deformation under load (in the sense of resulting stress and strain distributions) is perhaps the more important regulating factor, dependent on the physical properties of the bone tissue (e-modulus, elasticity, strength), the direction and amount of the applied forces, and also to a large extent by the geometry of the implant used. Parallel to the uncertainties concerning details of mineral formation at implant surfaces, it can be observed in implant literature that distinct implant types converge on similar screw-type implant systems. Screw-type implants of parabolic shape were shown to allow for a high primary congruence between the host site and the implant surface. Additionally, parabolic-shaped implants seem to impart advantages concerning the load transfer-related micromotion in the tissues adjacent to the implant surfaces. As such implants were used successfull under conditions of immediate loading, it must now be recognized that load related bone reactions at the implant interface may in combination with substrate effects (under distinct bone quality and quantity conditions) be responsible for an immediate osseointegration state. Therfore, it seems to be time to understand and define the term osseointegration in a new way.
